# The colonial origins of ethnic warfare: Re-examining the impact of communalizing colonial policies in the British and French Empires

**DOI:** 10.1177/00207152211023793

**Published:** 2021-06-16

**Authors:** Matthew Lange, Tay Jeong, Emre Amasyali

**Affiliations:** McGill University, Canada

**Keywords:** British Empire, colonialism, communalizing colonial policies, ethnic civil warfare, French Empire

## Abstract

Communalizing colonial policies (CCPs) include a variety of practices that recognize and institutionalize communal difference among colonized populations, and several qualitative analyses find that they promoted postcolonial ethnic conflict. In contrast, the few quantitative analyses that explore this issue focus on several mechanisms, make conflicting claims, and provide mixed results, thereby suggesting that CCPs do not have general effects. Yet the quantitative findings might be inaccurate for several reasons: Some use the identity of the colonizer as a proxy for CCPs, others measure a CCP but have small samples with limited variation in the focal independent variable, and all of these analyses are unable to explore whether CCPs affect ethnic conflict through different and competing mechanisms. To address these limitations, we create four ideal types of CCPs, gather data on a CCP that conforms to each ideal type, and test the relationships between CCPs and ethnic civil warfare onset using the set of former British and French colonies. We find that a discriminatory CCP is associated with high odds of ethnic civil war onset, especially shortly after independence. Alternatively, differentiating and accommodating CCPs lack general relationships with ethnic civil war onset, and an empowering CCP is negatively related to ethnic warfare in most models.

## Introduction

The ethnic civil war in Syria between Alawites, Sunnites, and Kurds and the ethnic civil war in Myanmar pitting Bamars against Rohingyas resulted in two of the greatest humanitarian crises of the 21st century. In addition to noting their shared destructiveness, commentaries on both wars note a similar historical determinant: colonialism (Dutta, 2019; Fildis, 2011; Polk, 2013; Sarkar, 2019). Such claims are not limited to Syria and Myanmar, as historians and social scientists provide evidence that colonialism also contributed to ethnic conflict in Sudan, Rwanda, Lebanon, Uganda, Sri Lanka, India, and elsewhere (De Silva, 1986; Idris, 2005; Lwanga-Lunyiigo, 1987; Mamdani, 2001, 2009; Newbury, 1983; Pollis, 1973; Tambiah, 1986). These works provide case studies and analyze how communalizing colonial policies (CCPs) that recognized and institutionalized communal divisions among colonized populations had influential effects on the conflicts. In Syria, for example, analyses find that community-based military recruitment, administration, and legislative representation during the colonial period—all of which institutionalized communal difference—contributed to the country’s violent postcolonial history (Bou-Nacklie, 1993; Fildis, 2011; Khoury, 1987; White, 2011).

Widespread claims that CCPs promoted ethnic conflict suggest a general pattern, and statistical analyses are ideal for highlighting such relationships. The growing number of cross-national statistical analyses of ethnic conflict, however, pays little attention to whether colonialism left violent legacies, and the few quantitative works that explore this issue provide weak and mixed results, thereby suggesting that no general pattern exists. Yet a variety of problems might affect the accuracy of these analyses. One major issue is measurement error, as most works assume that the British used CCPs much more than the French and employ British and French colonialism as proxies for the presence or absence of CCPs without actually testing whether CCPs were more concentrated in one empire. And the few works that measure CCPs analyze different samples of former British colonies, and the small number of cases might affect the results. Compounding this problem, analyses of British colonies potentially truncate variation in the focal independent variables, given common claims that CCPs were unusually common in the British Empire (Abernethy, 2000; Lange, 2017; Lange and Dawson, 2009). Finally, the authors of quantitative analyses propose a variety of competing mechanisms through which CCPs shape intercommunal relations, yet these analyses are unable to explore this possibility because they either use the identity of the colonizer as a proxy for CCPs or limit their analysis to one CCP.

In this article, we address these potential problems in different ways, thereby offering new insight into whether colonialism had any general effect on postcolonial ethnic conflict. To estimate the effects of several CCPs, we create a new dataset that measures the presence of four different CCPs and use statistical methods to test their relationships with the odds of ethnic civil war onset. We also analyze both British and French colonies in order to increase the number of cases and expand variation in the use of CCPs. Finally, we make conceptual contributions to the literature by developing four ideal types of CCPs based on the ways through which they shape intercommunal relations. We consider the extent to which actual CCPs conform to the ideal types and, in turn, explore whether different types of CCPs have contrasting relationships with postcolonial ethnic conflict.

The remainder of this article includes six sections. In the first, we define CCPs and review the literature on how they affect ethnic conflict, and the second section develops ideal types of CCPs and considers potential ways through which each type shapes ethnic conflict. In the third section, we focus on temporality and consider how colonial policies can shape postcolonial patterns of ethnic conflict. The fourth section presents a statistical analysis of former British and French colonies that explores relationships between different types of CCPs and the odds of ethnic civil war onset, and the fifth section applies past qualitative analyses to our findings for initial insight into mechanisms underlying the statistical results. Finally, we summarize our findings and consider their implications and limitations.

## CCPs and ethnic conflict: a review

The historical and social scientific literature on colonialism describes how colonial officials employed a variety of policies that increased the salience and strength of communal boundaries by explicitly recognizing the presence of multiple colonized communities. To date, scholars have not agreed upon a general term for these policies, and we refer to them as communalizing colonial policies, or CCPs. Indirect rule is one example of a CCP, as it recognizes communal difference in order to provide communities with a high degree of self-rule. While very different, colonial censuses that recognize communities and count their members are also CCPs. Other notable examples include language policies that recognize a variety of indigenous languages and policies that give preferential treatment to certain communities in key colonial institutions, such as the military.

The literature on colonialism argues that CCPs shaped intercommunal relations in important and enduring ways, although scholars disagree about the mechanisms through which CCPs affect ethnic conflict. The earliest analyses equate CCPs with “divide and rule” and claim that they increase intercommunal antipathy and hostilities by pitting communities against one another (Abernethy, 2000; Idris, 2005; Lange and Dawson, 2009; Mamdani, 2001; Morrock, 1973; Newbury, 1983; Pollis, 1973; Weber et al., 2016). These works note that CCPs were commonly discriminatory, thereby benefiting some communities at the expense of others. Those communities hurt by CCPs resented the communities that benefited from colonial policies, viewing them as the recipients of unfair advantages from an illegitimate colonial master. At the same time, communities benefiting from CCPs expected to retain their privileges and mobilized to protect them. And the co-presence of one community that was angry and resentful over colonial favoritism and another that assertively protected its advantages created a high risk of violence.

A related view focuses on whether CCPs created ranked or unranked systems of communal stratification, although scholars taking this position make competing claims. Similar to the divide-and-rule perspective, some argue that ranked systems promote ethnic conflict by increasing grievances over the relative status of communities (Horowitz, 1985; Lange, 2017). Complementing this view, others claim that unranked communal stratification limits the risk of ethnic civil warfare by recognizing and empowering communities in ways that promote communally inclusive power-sharing arrangements (Wucherpfennig et al., 2016). In contrast, however, others argue that unranked systems are more likely to increase the risk of ethnic conflict more than ranked systems because relative equality among communities promotes intercommunal competition (Blanton et al., 2001; Vogt, 2018).

A final position argues that CCPs increase the risk of ethnic conflict but claims that CCPs have this effect even when they are non-discriminatory and regardless of whether they promote ranked or unranked stratification systems. These works draw on the work of Tajfel (1970, 1974) and describe how colonial powers created ethnographic states that divided colonized peoples into communal categories in order to make them legible and, thereby, controllable (Cohn, 1987; Dirks, 2001; King, 1999; Mamdani, 2012; Wyrtzen, 2015). According to this view, CCPs promoted community-based cognitive frameworks, which, in turn, contributed to intercommunal discrimination, competition, and violence (Lieberman and Singh, 2012).

Most qualitative analyses of CCPs focus on British colonies (Christopher, 1988; De Silva, 1986; Idris, 2005; Lange, 2017; Mamdani, 2009; Pollis, 1973; Tambiah, 1986). This is not by coincidence, as several analyses note that the British Empire was exceptional in the extent to which it recognized and institutionalized communal difference among colonial subjects (Abernethy, 2000; Blanton et al., 2001; Crowder, 1968; Wucherpfennig et al., 2016). At the same time, these works argue that assimilationist policies and direct rule limited the use of CCPs in the French Empire. Based on this understanding, past statistical analyses assume that the British employed CCPs more than the French and use British colonialism as a proxy for CCPs and French colonialism as a proxy for their absence, thereby analyzing the relative prevalence of ethnic conflict in different empires to test of the impact of CCPs on ethnic conflict. These analyses provide very mixed results. Some find that British colonialism increased the risk of ethnic conflict relative to French rule (Blanton et al., 2001; Brunnschweiler and Bulte, 2009; Collier et al., 2009; Henderson, 2000; Lange and Dawson, 2009), other works find no colonial effect and downplay inter-imperial differences (Cederman et al., 2015; Collier and Hoeffler, 2002; Fearon and Laitin, 2003; Paine, 2019; Wimmer, 2018), and still others provide evidence that former French colonies are more prone to ethnic warfare (Wucherpfennig et al., 2016).

One major empirical problem shared by all of these quantitative analyses is the use of the identity of the colonizer as a proxy for CCPs. Colonizers did not employ CCPs uniformly throughout their empires, and these works cannot account for intra-imperial variation. Moreover, although many claim that the British used CCPs more than the French, past analyses of ethnic conflict do not actually test whether this is the case. A few analyses, in turn, argue that ethnic conflict differs between former British and French colonies because the French interfered in postcolonial politics in ways that constrained openings for ethnic conflict, not because of variation in the use of CCPs (Collier et al., 2009; Gregory, 2000). It is therefore uncertain what colonial proxies actually measure. Finally, past works focus on different aspects of CCPs and argue that they affect ethnic conflict through a variety of mechanisms, but colonial proxies provide a poor test of complex processes involving multiple and competing mechanisms.

Given these problems, comparative analyses of the impact of colonialism on ethnic conflict must develop measures of CCPs. A handful of analyses have already done so (Lange and Balian, 2008; Ray, 2018, 2019; Verghese, 2016; Wimmer, 2018). These works analyze either indirect rule or communalized police recruitment and use different samples of former British colonies. While helping to limit some of the problems caused by using proxies, these analyses also produce inconsistent results, and empirical shortcomings might contribute to these findings. Because many claim that CCPs were common in the British Empire, restricting the set to a sample of former British colonies potentially constrains variation in the use of CCPs, thereby promoting biased estimates. And compounding this problem, the small number of cases limits the strength of statistical insight. Finally, statistical analyses of only one CCP have great difficulty analyzing multiple and competing mechanisms.

## Ideal types of CCPs in the British and French Empires

We propose that the analysis of several CCPs in multiple empires can help to limit problems affecting past quantitative works and thereby improve general understandings of the impact of CCPs on ethnic conflict. To increase both the number of cases and variation in CCPs, we analyze former British and French colonies. And to explore the different mechanisms through which CCPs might affect ethnic conflict, we develop four ideal types of CCPs and analyze CCPs that conform to each type.

In an effort to expand the number of cases and variation in the use of CCPs, statistical analyses of CCPs might include all modern colonial empires. In this article, we use a more modest set of cases, choosing to limit our set to the British and French Empires for two reasons. The first is a concern for causal heterogeneity, as the colonies in different empires might be unique in ways that shape the impact of CCPs. Most notably, many overseas empires were very small, and the Spanish Empire declined more than a century before all other European overseas empires and long before CCPs became common practice. Second, a basic knowledge of the cases is necessary to interpret complex statistical patterns, and we have already completed considerable historical research on the British and French Empires.

Several characteristics, in turn, make the British and French Empires appropriate candidates for an analysis of CCPs. Both were the largest 19th and 20th century empires, thereby making possible a relatively large set of cases for analysis. Moreover, colonial powers increasingly used CCPs in the late 19th and early 20th centuries, and both powers maintained their vast empires until after the Second World War. Finally, many recognize that the British and French employed CCPs to different extents, thereby increasing variation in the use of CCPs.

The British and French used many different CCPs, and statistical analyses of multiple CCPs must therefore consider which CCPs to select. Two simple options are to select CCPs at random or based on data availability. Alternatively, scholars can note the theoretical focuses of past works and select CCPs that offer insight into these theories. A final strategy involves taking an inventory of CCPs and selecting CCPs that capture key aspects of variation. In this article, we employ the final strategy, although it overlaps with the third. Based on our review of the qualitative and quantitative literature on colonialism and ethnic conflict, we create four ideal types that focus on the different ways in which CCPs affect intercommunal relations—they differentiate between communities, accommodate the cultural practices of communities, empower communities, and discriminate between communities. And because the theories of past works focus on differentiation, empowerment, and discrimination, this strategy allows us to test the main mechanisms described in previous works.

**Figure 1.**
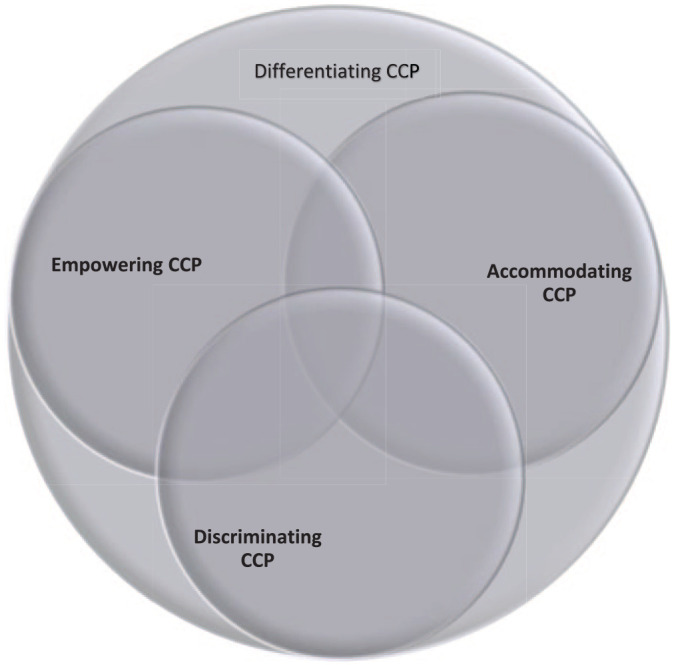
Venn diagram of types of CCP.

As Weber notes, ideal types exaggerate select aspects of phenomena, and researchers use ideal types to analyze the extent to which real-world cases conform to them (Kalberg, 1994). Social phenomena rarely match ideal types perfectly, however, and usually combine aspects of multiple ideal types. This is clearly the case with our ideal types, as CCPs can simultaneously differentiate, accommodate, empower, and discriminate. In fact, all CCPs must differentiate, suggesting that accommodating, empowering, and discriminating CCPs necessarily combine ideal types. Figure 1 uses a Venn diagram to depict the different possible combinations of the four ideal types. While recognizing their common combinatorial character, CCPs usually conform to one type more than another, and the type of CCP potentially shapes their effects on ethnic conflict.

Differentiating CCPs document communal difference by recognizing the presence of multiple communities. Because all CCPs do this, every CCP is a differentiating CCP. Some CCPs do more than simply differentiate between communities, however, and CCPs are closest to this ideal type when they have limited effects on empowerment, accommodation, or discrimination. One example of a highly differentiating CCP is the use of colonial censuses to collect information on different communities, something that recognizes the existence of different communities and institutionalizes communal difference while usually having little direct, clear, or systematic effects on empowerment, accommodation, or discrimination.

The main effect of a differentiating CCP is on the presence and strength of communal cognitive frameworks. That is, formal recognition of the presence of communities causes people to pay attention to communities and perceive the world in terms of communities. In his study of the Indian colonial census, for example, Cohn (1987) finds that collecting information on caste greatly increased the salience of caste. From a Tajfelian perspective, differentiating CCPs promote intercommunal competition and discrimination and therefore increase the risk of ethnic conflict, and Cohn’s analysis supports this view by showing that the strengthening of caste categories contributed to competition and conflict. Alternatively, a more pluralist position suggests that formal recognition of communities can help to manage ethnic diversity by declaring all communities members of the nation and thereby limiting the perceived or real exclusion of communities. We believe that both positions highlight competing mechanisms that can influence ethnic conflict and therefore hypothesize that differentiating CCPs have little or no general effect.

Discriminating CCPs recognize communities in order to privilege particular communities more than—and commonly at the expense of—other communities. Common examples include stacking the colonial military with particular communities, using only one of several vernaculars in colonial education, and providing only certain communities with political autonomy. The divide-and-rule position focuses explicitly on discrimination and claims that it promotes ethnic conflict by increasing intercommunal antipathy and, thereby, competition. The literature on ranked and unranked stratification systems also considers the influence of discriminatory policies, although some taking this position consider different and competing mechanisms. Whereas many focusing on ranked and unranked stratification systems concur with the divide-and-rule position, others ignore the impact of discriminatory policies on intercommunal antipathy and suggest that discriminatory policies deter ethnic conflict by limiting communal competition. Similar to the divide-and-rule perspective, we focus on the antipathy caused by discriminatory CCPs and hypothesize that highly discriminatory CCPs increase the risk of intercommunal conflict. Communities that are negatively affected by these policies target communities that benefited from the discriminatory CCPs with anger and resentment, viewing them as little more than colonial “stooges.” At the same time, those communities benefiting from the discriminatory CCPs mobilize to protect their privileged positions. And we hypothesize that the resulting confrontation creates a high risk of ethnic conflict.

Accommodating CCPs recognize communal difference in order to provide communities with public goods and services that accommodate cultural difference. For example, colonial elementary schools commonly offered primary education in a variety of different vernaculars in order for students to begin their educations in their mother tongues. Similarly, colonizers sometimes accommodated cultural difference by offering different community-based systems of family and marriage law. Although CCPs frequently deal with multicultural accommodation, the literature on colonialism and ethnic conflict pays little attention to this type of CCP.

The different perspectives of colonialism and ethnic conflict can be applied to accommodating CCPs and suggest that this type of CCP has mixed effects. Accommodating policies provide communities with valuable resources, including the provisioning of services that respect cultural differences and providing communities with a certain symbolic status. From the pluralist perspective, such provisioning and status help to promote more communally inclusive political systems, which many find deters ethnic warfare (Wimmer et al., 2009; Wucherpfennig et al., 2016). At the same time, accommodating CCPs heighten intercommunal differences and can promote competition and discrimination. In particular, accommodating CCPs push communities to pursue their particular interests in the formal political arena, something that communalizes politics and potentially creates a heightened risk of ethnic conflict. Believing that both mechanisms are influential, we hypothesize that accommodating CCPs have no general effect on ethnic conflict.

Empowering CCPs recognize communal difference in order to provide communities with formal power and autonomy. Thus, whereas accommodating CCPs provide communities with culturally targeted services, empowering CCPs commonly provide communities with the power to provide their own services. Indirect rule, which allows communities a high degree of self-rule, is a notable example. For instance, the British controlled the Buganda, Acholi, and many other communities in colonial Uganda by recognizing “traditional” chiefs and allowing them to run local administrative and legal systems.^1^ British colonialism therefore endowed communal elites in Uganda with considerable political power in the name of their communities.

Similar to differentiating and accommodating CCPs, we argue that empowering CCPs have mixed effects on ethnic conflict. Like accommodating CCPs, empowering CCPs can communalize politics and thereby promote intense intercommunal competition in the formal political realm, something that promotes intercommunal violence (Lange, 2017). At the same time, empowering CCPs can deter ethnic conflict by promoting inclusive power-sharing arrangements. As Wucherpfennig et al. (2016) note, communal empowerment forces states to be responsive to communities and share power with them, and both deter ethnic warfare.

## CCPs and the timing of postcolonial ethnic conflict

Over the past two decades, social scientists paid increasing attention to temporality and the role of time in causal processes. Temporality is a crucial issue for studies of colonial legacies, as researchers must consider how the impact of colonialism can extend beyond the colonial period to shape postcolonial processes. In this section, we consider issues of temporality and how CCPs can shape postcolonial patterns of ethnic conflict.

Most analyses of colonial legacies focus on path dependence, a temporal concept suggesting that past events have lingering effects (Mahoney, 2000; Pierson, 2000). During path-dependent processes, some transformative effect structures social relations, and different mechanisms reinforce these structures long after the initial transformative event ends, thereby allowing the event to have long-lasting effects. Although the literature on colonialism and ethnic conflict ignores path dependence, its arguments are implicitly path-dependent. Those claiming that CCPs promoted ethnic conflict suggest that the antagonisms, competition, and divisions resulting from colonialism continue after independence and thereby have long-term effects. Similarly, those works that focus on the suppressive effects of CCPs recognize that inclusive power-sharing arrangements become institutionalized and persist, thereby making possible colonial legacies.

While agreeing that such path dependence is common, we hypothesize that the impact of CCPs on the risk of ethnic conflict is greatest immediately following independence and decreases thereafter. One clear reason for a more punctuated postcolonial impact is that postcolonial social change is inevitable and weakens path-dependent effects. To the extent that postcolonial change reduces the long-term influence of colonialism, colonial legacies should therefore be strongest immediately after independence and weaken over time thereafter.

Postcolonial ethnic conflict is likely concentrated around independence for a second reason. As Wimmer (2013) finds, independence promotes conflict over the control of the postcolonial state and the communal character of the postcolonial nation, and the risk of ethnic warfare is therefore particularly high in the years right after independence. Thus, to the extent that CCPs communalize politics and create intercommunal antagonisms over recognition, rights, privileges, and power, places that experienced CCPs are most likely to have a heightened risk of ethnic conflict during the immediate postcolonial periods. And because we hypothesize that discriminatory CCPs are most likely to promote intercommunal hostilities, we argue that ethnic conflict is particularly prevalent shortly after independence in places with this type of CCP.

## Statistical analysis: CCPs and ethnic civil war onset

Having reviewed the literature, presented ideal types of CCPs, and considered how they affect postcolonial ethnic conflict, we now run a series of logit regressions to estimate the impact of CCPs on the odds of ethnic civil war onset. As part of this analysis, we explore whether the relationships between CCPs and ethnic conflict change over time. For these analyses, we gathered panel data ranging from 1946 to 2010 with the country-year as the basic unit of observation.

Our set includes all formal British and French overseas colonies that have more than one million inhabitants. When a colony was simultaneously controlled by multiple powers, we recognize the colonizer as the foreign power that ruled over the largest population. Alternatively, when a colony was ruled by multiple colonizers in succession, we recognize the last power that formally colonized the region for at least 10 consecutive years as the colonizer. Our exclusion of countries with less than one million people follows past statistical analyses of ethnic violence, which remove these cases either because of missing data or over concerns about causal heterogeneity.^2^ And because we define colonialism as formal imperial control of domestic and foreign policies in overseas territories, we exclude informal colonies, internal colonies, and protectorates in which the imperial power simply controlled foreign policy. Based on these criteria, our set includes 34 British colonies and 24 French colonies.

### Focal independent variables

We use four indicators of CCPs as focal independent variables. These include measures of communal census categories, indirect rule, vernacular primary education, and communal legislative representation. An analysis of these policies offers insight into whether different types of CCP have contrasting effects.

Communal census categories measure the extent to which colonial censuses formally recognize communal difference by collecting data on different types of communities, and this practice conforms closely to the differentiating CCP ideal type. In colonial India, for example, officials used censuses to recognize and count a great diversity of communities based on language, religion, case, tribe, and race, and this practice helped to formalize, strengthen, and—at times—create communal boundaries (Cohn, 1987; Dirks, 2001; King, 1999). In many other colonies, however, censuses either ignored all communal categories or gathered data on only one or two types of community.

To measure the extent to which colonizers used censuses to recognize communal difference, we use data from Lieberman and Singh (2017) on whether censuses gathered information on caste, linguistic, racial, religious, and tribal categories and calculate the largest number of types of categories ever collected in a colonial census. Scores range from 0 (no types of communal categories collected) to 5 (caste, ethnic, racial, religious, and tribal categories all collected), with higher scores suggesting that colonial powers used censuses as a CCP. We score this variable as 0 for cases in which the colonizers never completed censuses. For the analysis, we use communal census categories as a continuous variable and standardize it.

Our second focal independent variable measures the extent of indirect colonial rule, which is a clear indicator of an empowering CCP. The literature on indirect rule notes that this form of colonialism provides communities with a high degree of self-rule, as it gives indigenous authorities control over local administrative and legal systems. To measure the extent of indirect rule, we use data from Lange (2009) on the proportion of the total colonial court cases heard in customary courts presiding over both civil and criminal matters and ruled by traditional authorities who also controlled local administrations. The variable is continuous and ranges from 0 to 1, with higher numbers suggesting a greater use of indirect rule.^3^ To facilitate interpretation, we standardize this variable for all cases.

Notably, Lange (2009) only provides data for British colonies, and there is no equivalent of customary courts in French colonies. Although many recognize that the French employed indirect rule, the French form did not attempt to empower communities by providing them with their own local administrations and legal institutions (Collier, 1982; Crowder, 1968; Lange 2009; Müller-Crepon, 2020). Instead, the French sometimes used traditional authorities at the state level as powerless puppets in an effort to transfer customary legitimacy to the colonial state. Most commonly, the French incorporated indigenous administrators into colonial bureaucracies at the local level and placed them under the control of French officials, and the indigenous administrators followed the orders of their French bosses and rarely had any traditional authority. Customary tribunals in French colonies, in turn, conformed more closely to accommodating CCPs than empowering CCPs, as they only dealt with family and marriage law and the indigenous judges had no administrative powers. Given these important differences, we therefore score the extent of indirect rule as 0 for all French colonies.

For our third indicator, we use communal legislative representation as an example of a discriminatory CCP. This CCP involves providing communities with legislative representation either by reserving seats for them, creating separate communal electorates, or providing communities with their own assemblies. Examples of communal legislative representation include providing Muslims with special electorates in colonial India and reserving seats for Tamils in colonial Sri Lanka. Although communal legislative representation is an empowering CCP, it is also a highly discriminatory CCP in that it only gives special legislative representation to select communities. Communal legislative representation, in turn, is a particularly appropriate example of a discriminating CCP because it is formal and all communities are cognizant of it, and this awareness is necessary for discrimination to promote popular grievances and antipathy.

To measure the presence of communal legislative representation, we gather information from primary and secondary sources to create a dichotomous variable measuring whether communal legislative representation was present at any time during the colonial period. For a score of 1, the colonizer must have employed communal legislative representation in colony-level legislative assemblies at some time, and this representation must have privileged certain communities relative to others. Because of our interest in indigenous divisions, we score cases as 0—no communal legislative representation—if legislatures only gave special representation to non-indigenous peoples, such as White settlers. See Appendix 1 for information on our coding of individual cases for this variable.

The provisioning of colonial education in indigenous vernaculars is a clear example of an accommodating CCP, and we use a dichotomous indicator of whether colonizers provided primary education in multiple vernaculars to measure the presence of this CCP. We use data on the language of education from Albaugh (2014) for British and French colonies in sub-Saharan Africa (as well as Algeria) and gathered data from primary and secondary sources for all other British and French colonies. See Appendix 1 for information on the coding of this variable.

Table 1 provides a correlation matrix of the four CCP indicators, and the results suggest that places with one CCP were more likely to have others. The use of multiple vernaculars in primary education has particularly strong relationships with communal census categories and communal legislative representation. Table 2 lists the average scores of our indicators of CCPs by the colonizer (see Appendix 2 for the scores of each colony in our set). Although showing considerable intra-imperial variation, the data highlight clear inter-imperial patterns, with CCPs being more concentrated in British colonies. The British collected two and one-half times as many types of communal categories on colonial censuses, were three times more likely to have communal legislative representation, and were 15 times more likely to provide primary education in multiple vernaculars. Finally, 39 percent of all court cases in former British colonies were heard in customary courts run by indigenous authorities presiding over both criminal and civil cases and running local administrations, whereas there were no such courts in French colonies. The evidence therefore supports past claims that CCPs were more common in the British Empire than the French Empire. This general difference, in turn, offers evidence of considerable variation in the use of CCPs in the British and French Empires, making possible a systematic analysis of the relationships between CCPs and ethnic conflict.

**Table 1.** Correlation among CCPs.

	1	2	3	4
1. Legislative representation	1.00			
2.Multiple education vernaculars	0.72	1.00		
3.Census categories	0.45	0.79	1.00	
4. Extent of indirect rule	0.41	0.47	0.39	1.00

CCP: communalizing colonial policy.

1 and 2 are binary, 3 and 4 are coded as continuous. Correlations between a binary and a continuous variable show biserial correlation. Correlations between two binary variables show tetrachoric correlation. Correlations were computed with the “polycor” package in *R* (Fox, 2019).

**Table 2.** CCPs by colonizer.

	British			French			Difference	
	*n*	Mean	*SD*	*n*	Mean	*SD*	*t*	*p*
Census categories	27	3.15	1.32	23	1.30	1.26	–5.04	0.00
Multiple education vernaculars	27	0.59		23	0.04		4.09	0.00
Communal legislative representation	27	0.52		23	0.17		2.53	0.01
Extent of indirect rule	27	0.41	0.31	23	0.00	0.00	–6.90	0.00

CCP: communalizing colonial policy.

## Dependent and control variables

To measure ethnic conflict, we focus on ethnic warfare, and our dependent variable measures whether an ethnic civil war began in a given postcolonial year. For this, we use data from the Ethnic Armed Conflict dataset (Wimmer et al., 2009). This dataset includes the dates and locations of all ethnic civil wars that occurred between 1946 and 2010.

Because our data on ethnic civil warfare begin in 1946 and because several British colonies—Australia, Canada, Egypt, Iraq, New Zealand, South Africa, and the United States—and one French colony—Haiti—received their independences well before this date, some cases lack complete data for all post-independence years, something that might bias our results. The cases with early independences were also unique in ways that could bias our analysis in additional ways: They gained their independences decades before other colonies, most were either settler or plantation colonies, others were colonized for short periods, a few experienced informal American rule, and all of these characteristics might have shaped long-term patterns of ethnic warfare in unique ways. We therefore complete analyses using both the full set of cases and the set of cases with dates of independence after 1945. The results are substantively identical regardless of the set, and we only present the findings using the more restricted set.

To account for other factors that might affect ethnic civil war onset, we include 11 control variables. The first measures the number of previous ethnic civil wars in a country. As the impact of colonialism potentially varied over time, we include the number of years since independence as a second control variable. For some models, we also create interaction terms multiplying the time since colonial independence by the different types of CCP to test our hypotheses about the timing of ethnic conflict.

The next four variables control for factors that preceded colonialism and potentially confound relationships between CCPs and postcolonial ethnic warfare if omitted from the analysis. The first measures a country’s latitude. As noted by Alesina et al. (2003), latitude is associated with ecological conditions that shape long-term trajectories of development and communal diversity. Among our set of cases, for example, the correlation between latitude and contemporary ethnic fractionalization is ‒0.49, whereas the correlation between latitude and log of per capita gross domestic product (GDP) is 0.40. Precolonial ethnic diversity and development, in turn, might have shaped both CCPs and postcolonial ethnic warfare. Our second historical control measures the natural log of mountainous terrain as a percentage of total territory, which past analyses find increases the ability of anti-state combatants to fight civil wars (Fearon and Laitin, 2003). At the same time, this environmental condition might facilitate anticolonial resistance, and resistance could contribute to the use of CCPs. Our third historical control measures the presence of a large and long-standing precolonial state. Paine (2019) and Ray (2019) argue that precolonial states have long-term effects on ethnic warfare by promoting conflict between communities with and without precolonial states.^4^ Yet this social environment was ideal for divide-and-rule-style policies and might have promoted the use of CCPs. To measure the level of precolonial statehood, we use data from Borcan et al. (2018) on the extent to which there was a long-standing and autonomous precolonial state that controlled the same territory as a contemporary state. The variable ranges from 0 to 50 and measures 50-year periods, and we average the scores for all periods between 1001 and 1700 AD. Finally, we include a measure of the natural log of total population in 1900. Total population is strongly and consistently related to ethnic warfare in past analyses, and population size might have affected the use of CCPs. Notably, we standardize both latitude and state history in the statistical analyses.

Our next set of controls includes several variables commonly used in past statistical analyses of ethnic warfare: ethnic fractionalization (Fearon, 2003),^5^ the natural log of per capital GDP (Feenstra et al., 2015), the natural log of total population (Feenstra et al., 2015), the percentage of the population excluded from formal politics based on their ethnicity (Wimmer et al., 2009),^6^ and level of democracy (Marshall and Gurr, 2020). For the analysis, we standardize ethnic fractionalization and center log GDP pc and log population at the mean. Because of a strong relationship between population in 1900 and later population size, we remove the variable measuring population size in 1900 when including yearly measures of total population. Moreover, our measure of democracy is never significant, has no substantive impact on the estimated effects of our focal independent variables, and removes ethnic group-years and ethnic civil wars from our set because of missing data. In order to present results based on the full set, we do not include democracy in the models. Finally, some argue that CCPs—and especially indirect rule—affect ethnic violence through their impact on political exclusion, and the inclusion of the measure of political exclusion might therefore underestimate any effect of CCPs. We therefore run models with and without this variable. The estimated coefficients are substantively identical for the four measures of CCPs regardless of whether we include or exclude political exclusion. We only present the results of models without this variable.

As a final control, we include a dummy variable measuring the identity of the colonizer, with 1 measuring British rule and 0 measuring French rule. We include this variable to see if the identity of the colonizer either influences the relationships between CCPs and ethnic civil warfare or affects ethnic warfare in ways other than through the CCPs that we analyze. See Table 3 for the descriptive statistics of the control variables.

**Table 3.** Descriptive statistics of control variables.

	*n*	Mean	*SD*	Min.	Q25	Q50	Q75	Max.
Latitude	2617	0.19	0.11	0.01	0.09	0.17	0.27	0.39
Log mountainous terrain	2617	1.69	1.39	0	0.07	1.69	2.82	4.42
Precolonial statehood	2617	21.35	15.56	0	7.14	24.11	35.49	48.04
Log population 1900	2617	0.28	1.54	–2.40	–0.73	0.10	1.18	5.60
Proportion excluded	2617	0.20	0.25	0	0	0.10	0.38	0.97
Log population	2617	1.97	1.46	–0.97	0.94	1.88	2.77	7.12
Log GDP pc	2617	0.51	1.03	–1.75	–0.24	0.22	1.11	3.91
Ethnic fractionalization	2617	0.61	0.23	0.04	0.48	0.64	0.79	0.95
Number of previous wars	2617	0.65	1.42	0	0	0	1	10
Years since independence	2617	26.04	15.76	0	13	26	39	64
Extent of democracy	2543	–1.38	6.43	–10	–7	–4	5	10

GDP: gross domestic product.

Raw scale before standardization or centering.

## Analysis

Employing these variables, we run a series of logit regressions to estimate the impact of CCPs on the odds that an ethnic civil war begins in a given postcolonial year. For this analysis, we use cluster-robust standard errors to account for within-country dependence among observations. To facilitate the interpretation of our findings, we present all variable coefficients as odds ratios and provide 95-percent confidence intervals. Statistical significance, however, is a contested issue for our analysis. As our dataset consists of all former British and French colonies that gained their independences after 1945, our set is a clear example of an “apparent population,” which some authors argue is unsuitable for significance testing (Berk et al., 1995). If accepting this view, our statistical findings are best interpreted as merely *describing* the relationships between variables as they played out in actual history (Bollen, 1995: 464). Yet, one can also view British and French colonies as one random sample of many different samples that could have been generated by the same (putative) underlying macro-historical mechanisms, and significance levels are relevant if accepting this view (Bollen, 1995). In this article, we follow disciplinary norms by reporting significance levels but also consider the magnitude of coefficients even when they are insignificant.

The results in Table 4 copy the analytic strategies of previous works by using a British colonial dummy as the focal independent variable and exploring whether ethnic warfare varied by the identity of the colonizer. In addition to the British dummy variable, Model 1 includes the controls for time since independence, previous conflict, and the historical factors, and Model 2 adds the common controls to Model 1 (while removing the variable measuring the natural log of total population in 1900). In both models, the odds ratios of the British dummy are close to 1 and insignificant, thereby offering evidence that the odds of postcolonial ethnic civil war onset were similar for British and French colonies.

**Table 4.** Logit regression on ethnic civil war onset by colonizer, 1946–2010.

	(1)	(2)	(3)
British colony	0.782 (0.354, 1.731)	1.009 (0.466, 2.184)	3.449^*^ (1.111, 10.714)
Number of previous wars	1.251^*^ (1.015, 1.541)	1.161^+^ (0.987, 1.367)	1.239^*^ (1.050, 1.462)
Years since independence	0.992 (0.973, 1.012)	1.005 (0.984, 1.027)	1.029^+^ (0.999, 1.060)
Latitude	0.868 (0.648, 1.163)	1.417^*^ (1.022, 1.964)	1.346^+^ (0.989, 1.834)
Log proportion mountainous	1.156 (0.922, 1.449)	1.048 (0.836, 1.313)	1.040 (0.844, 1.281)
State history	1.053 (0.691, 1.604)	1.614^*^ (1.065, 2.448)	1.610^*^ (1.097, 2.363)
Log population 1900	1.266^**^ (1.059, 1.513)		
Log population		1.078 (0.886, 1.311)	1.087 (0.921, 1.283)
Log GDP pc		0.487^**^ (0.283, 0.836)	0.517^**^ (0.326, 0.821)
Ethnic fractionalization		2.344^***^ (1.553, 3.537)	2.242^***^ (1.532, 3.280)
British colony × years since independence			0.954^**^ (0.924, 0.985)
Constant	0.023^***^ (0.012, 0.042)	0.012^***^ (0.006, 0.025)	0.006^***^ (0.002, 0.016)
N group-years	2617	2617	2617
N war onset	63	63	63
AIC	565.24	546.28	541.69

AIC: Akaike information criterion; GDP: gross domestic product.

+ *p* < 0.1; ^*^p < 0.05; ^**^p < 0.01; ^***^p < 0.001.

The odds ratios of years since colonial independence is also near 1 and insignificant in both models, thereby suggesting that ethnic civil wars are not concentrated around the immediate postcolonial period. Previous works suggest that ethnic civil wars in former French colonies were rare until the 1990s because France interfered in postcolonial politics in ways that limited openings for civil warfare (Collier et al., 2009; Gregory, 2000). To explore this possibility, we add an interaction term multiplying British colonialism by years since independence in Model 3 of Table 4. The results estimate that the odds of ethnic civil war onset were three and one-half times greater at independence among former British colonies but decreased thereafter. Alternatively, the coefficient of years since independence offers evidence that the odds of ethnic civil war onset increased over time in former French colonies.

Table 5 provides a more direct test of the impact of CCPs by substituting our variables measuring different CCPs for the British dummy variable. The first two models in Table 5 use the same control variables as the first two models in Table 4, and Model 3 of Table 5 adds the British colonial dummy variable to Model 2. The results show that the relationships between the CCPs and the odds of ethnic civil war onset vary considerably. The estimated odds ratios of census categories are slightly below one but insignificant in all models, suggesting that differentiating CCPs do not influence ethnic warfare. Alternatively, the odds ratios of indirect rule are below one in all three models and significant in the last two, thereby providing evidence against our hypothesis that empowering CCPs have no general effect but supporting Wucherpfennig et al.’s (2016) claim that empowering CCPs deter ethnic warfare by promoting inclusive power-sharing arrangements. The coefficients of multiple vernacular languages are near one in Model 1, above one in Models 2 and 3, but insignificant in all models. Finally, communal legislative representation, which is a highly discriminatory CCP, consistently has odds ratios that are well above one and significant. The coefficients estimate that the odds of ethnic civil war onset are between 280 and 460 percent greater in places with communal legislative representation, thereby suggesting a considerable general effect.

**Table 5.** Logit regression of ethnic civil war onset on CCPs, 1946–2010.

	(1)	(2)	(3)
Census categories	0.882 (0.494, 1.577)	0.755 (0.405, 1.410)	0.648 (0.334, 1.256)
Multiple education vernaculars	0.996 (0.419, 2.366)	1.776 (0.539, 5.851)	1.680 (0.562, 5.024)
Legislative representation	2.832^*^ (1.143, 7.018)	4.573^**^ (1.819, 11.494)	4.267^**^ (1.683, 10.818)
Extent of indirect rule	0.757 (0.532, 1.075)	0.536^**^ (0.353, 0.813)	0.400^**^ (0.218, 0.734)
Number of previous wars	1.189^+^ (0.968, 1.459)	1.050 (0.889, 1.239)	1.009 (0.868, 1.172)
Years since independence	0.993 (0.975, 1.012)	1.013 (0.987, 1.038)	1.016 (0.991, 1.042)
Latitude	0.726^*^ (0.529, 0.996)	1.120 (0.827, 1.516)	1.219 (0.886, 1.676)
Log proportion mountainous	1.195 (0.935, 1.529)	1.031 (0.820, 1.295)	1.034 (0.811, 1.319)
State history	0.860 (0.558, 1.325)	1.366 (0.801, 2.329)	1.425 (0.782, 2.597)
Log population 1900	1.326^**^ (1.118, 1.572)		
Log population		1.174^+^ (0.980, 1.406)	1.186^+^ (0.983, 1.431)
Log GDP pc		0.381^**^ (0.213, 0.681)	0.359^***^ (0.208, 0.619)
Ethnic fractionalization		2.972^***^ (1.663, 5.312)	3.716^***^ (1.816, 7.604)
British colony			2.685 (0.609, 11.846)
Constant	0.013^***^ (0.007, 0.022)	0.004^***^ (0.001, 0.010)	0.002^***^ (0.001, 0.008)
N group-years	2617	2617	2617
N war onset	63	63	63
AIC	566.43	540.44	540.40

CCP: communalizing colonial policies; GDP: gross domestic product; AIC: Akaike information criterion.

+ *p* < 0.1; ^*^p < 0.05; ^**^p < 0.01; ^***^p < 0.001.

Our finding that discriminating CCPs promote ethnic civil warfare potentially has implications on how to interpret the results of differentiating, accommodating, and empowering CCPs. Although census categories, vernacular education policies, and indirect rule conform most closely to differentiating, accommodating, and empowering ideal types, respectively, colonizers might have used them in a discriminatory fashion in some cases. And if discriminatory uses of CCPs promote ethnic warfare, these CCPs likely have mixed effects. Unfortunately, we are unable to explore this possibility statistically.

Table 6 explores whether the estimated impact of the CCPs varied over time by adding interaction terms multiplying each CCP indicator by years since independence. All models presented in the table include the full set of controls, with the first four models only including one interaction term at a time whereas the fifth model includes all four interaction terms simultaneously. Although including all control variables, we only present the results for the CCPs, years since independence, and the interaction terms to limit the size of the tables.

**Table 6.** Logit regression including CCP and timing interaction, 1946–2010.

	(1)	(2)	(3)	(4)	(5)
Legislative representation	17.543^***^ (4.829, 63.729)	4.867^**^ (1.874, 12.635)	4.047^**^ (1.626, 10.071)	4.831^***^ (1.926, 12.120)	17.498^***^ (3.442, 88.953)
Census categories	0.751 (0.429, 1.316)	0.792 (0.413, 1.520)	0.739 (0.419, 1.304)	0.700 (0.396, 1.237)	0.383^**^ (0.200, 0.734)
Multiple educ. vernaculars	1.262 (0.454, 3.509)	1.378 (0.483, 3.933)	3.320 (0.742, 14.850)	1.337 (0.469, 3.811)	3.226 (0.754, 13.797)
Extent of indirect of rule	0.453^**^ (0.253, 0.811)	0.403^**^ (0.207, 0.783)	0.437^**^ (0.238, 0.802)	0.636 (0.296, 1.365)	0.569^+^ (0.299, 1.083)
Years since independence	1.040^**^ (1.011, 1.071)	1.012 (0.986, 1.039)	1.023^+^ (0.997, 1.051)	1.012 (0.986, 1.038)	1.051^**^ (1.014, 1.089)
Legislative representation × years since independence	0.950^**^ (0.919, 0.982)				0.952^*^ (0.915, 0.990)
Census categories × years since independence		0.996 (0.981, 1.012)			1.023^*^ (1.000, 1.047)
Multiple educ. vernaculars × years since independence			0.968 (0.930, 1.007)		0.964 (0.916, 1.014)
Extent of indirect rule × years since independence				0.982^*^ (0.967, 0.998)	0.989^+^ (0.978, 1.000)
Constant	0.001^***^ (0.0004, 0.003)	0.002^***^ (0.001, 0.008)	0.002^***^ (0.001, 0.005)	0.002^***^ (0.001, 0.006)	0.001^***^ (0.0002, 0.003)
N group-years	2617	2617	2617	2617	2617
N war onset	63	63	63	63	63
AIC	535.29	541.7	538.87	537.83	536.94

CCP: communalizing colonial policies; AIC: Akaike information criterion.

+ *p* < 0.1; ^*^p < 0.05; ^**^p < 0.01; ^***^p < 0.001.

**Figure 2.**
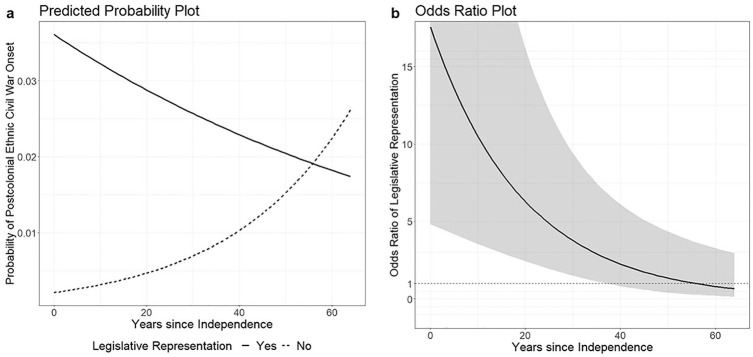
Interaction between communal legislative representation and years since independence (Table 6, Model 1): (a) Predicted probability plot. (b) Odds ratio plot.

The odds ratios and significance levels of communal legislative representation are large and significant in all models, but they are extremely large in the models including the interaction term multiplying years since independence by communal legislative representation. These results estimate that a history of communal legislative representation increased the odds of ethnic civil war onset at independence by 17-fold. Figure 2 plots the estimated interaction effects between legislative representation and years since independence using Model 1 in Table 6. Figure 2(a) visualizes the predicted probability of postcolonial ethnic civil war onset by years since independence for countries with and without a history of communal legislative representation.^7^ The figure shows that having a history of communal representation is associated with a much higher risk of ethnic civil war onset at the time of independence and that this difference decreases over time. Figure 2(b) shows the odds ratio of legislative representation by time. Given that the probabilities are generally very low (<0.03), the odds ratio serves as a close approximation of the risk ratio, which is the ratio between the bold and dotted lines in Figure 2(a). As indicated by the shaded 95-percent confidence intervals in Figure 2(b), the odds ratio is significantly above 1 until more than 30 years after independence, showing that a history of colonial communal representation is associate with elevated odds of ethnic civil war onset long after independence. Overall, the findings support our hypotheses that discriminatory CCPs promote ethnic conflict and that these effects were especially concentrated shortly after independence.

In contrast, the results in Table 6 offer little evidence that the effects of indirect rule, census categories, or vernacular education were greater during the early independence period. The results for census categories are inconsistent, with Model 2 estimating little or no effect either at independence or thereafter but Model 5 offering evidence that the use of communal census categories reduced the risk of ethnic civil war onset at independence but that this effect weakened over time. The coefficients of multiple educational vernaculars are well above one but insignificant in Models 2 and 5, and the coefficients of the interaction term multiplying this variable by time since independence are below one but insignificant in both models. Finally, the coefficients of indirect rule and the interaction term multiplying indirect rule by time since independence are all below one in Models 4 and 5, thereby offering evidence of similar relationships over time.

## Mechanisms linking discriminatory CCPs and ethnic warfare

The statistical findings strongly and consistently support our hypothesis that discriminatory CCPs promote ethnic warfare, especially in the decades following independence. In contrast to our hypothesis, however, we find that indirect rule is associated with a reduced risk of ethnic civil warfare. Yet our statistical analysis cannot highlight the mechanisms through which CCPs shape intercommunal relations, and we believe that such insight must supplement statistical analyses for a convincing causal account. Although a qualitative assessment of causal mechanisms is beyond the scope of our analysis, in this section, we offer initial insight into underlying mechanisms linking discriminatory CCPs to ethnic warfare by applying past qualitative findings to our analysis, thereby offering an informed mechanistic interpretation of the statistical results. We do not explore mechanisms linking indirect rule to limited ethnic warfare because we were unable to find any qualitative works on this topic.

Relative to other discriminatory CCPs, little attention has been paid to the influence of communal legislative representation on ethnic conflict, although case studies of India, Myanmar, Sri Lanka, Lebanon, and Syria all suggest that communal legislative representation contributed to ethnic warfare. These works offer evidence that such representation communalizes politics while, at the same time, promoting intense competition over the control of the postcolonial state and the form of the postcolonial nation. In India, Breuilly (1994: 75–82), Metcalf and Metcalf (2006: 160–161), and Tudor (2013) offer evidence that the provisioning of special legislative representation for Muslims contributed to the communalization of Indian politics and, ultimately, demands for the division of India into separate nation-states. The eventual separation of India, in turn, had spillover effects contributing to ethnic civil wars, most directly in the Kashmir region. In her exceptional analysis of colonial politics, Russell (1982) makes similar claims for Sri Lanka. She finds that debate and conflict over the use of communal legislative representation promoted the rise of ethnic-based political parties and Sinhalese ethnic nationalism, both of which contributed to the country’s bloody civil war (see also De Silva, 1986). In a similar way, Lange et al. (forthcoming) provide evidence that the provisioning of Karens with special legislative representation in colonial Myanmar communalized politics and had influential effects on the rise and strength of Karen and Bamar ethnic nationalism, both of which contributed to a six-decade-long civil war. Finally, the French only institutionalized formal communal legislative representation in Lebanon and Syria,^8^ and several past works find that communal representation contributed to ethnic civil wars in both places by exacerbating communal political competition (Fildis, 2011; Hourani, 1954; Khoury, 1987; White, 2011).

Communal legislative representation is only one example of a discriminatory CCP, and it is possible that other discriminatory CCPs had similar effects. In fact, one potential problem with our findings on census categories, vernacular education, and indirect rule is that these policies can be used discriminatorily. A variety of cases, in turn, offer evidence that discriminatory uses of indirect rule contributed to ethnic warfare. In the regions that are presently Northeastern India, Southeastern Bangladesh, and Northern Myanmar, for example, the British ruled several minority communities indirectly while ruling larger neighboring communities more directly, thereby advantaging minority communities relative to other communities. After independence, postcolonial states sought to weaken the autonomy of these minority communities, sparking backlashes that contributed to ethnic civil wars. Combined with our statistical findings, this suggests that indirect rule has opposing effects depending on whether it is used discriminatorily but that colonial officials usually employ indirect rule in a non-discriminatory fashion.

Discriminatory educational language policies might also contribute to ethnic warfare in similar ways. For example, the exclusive use of Arabic in Sudanese schools privileged a relatively small group of Nilotic Arabs and marginalized non-Arab communities, and analyses find that this marginalization contributed to ethnic civil warfare (Idris, 2005; Sharkey, 2008). Yet other CCPs also appear to have interacted with linguistic policies and likely had more powerful effects on ethnic warfare in Sudan. Due in part to educational inequalities, the British stacked the colonial military and administration with Arabs, thereby promoting communal inequalities and placing Arabs in a position to dominate the postcolonial state. At the same time, the British gave non-Arabs in Southern Sudan considerable autonomy through indirect rule and created a bifurcated administrative structure that ruled Northern and Southern Sudan separately. Thus, when postcolonial governments pursued Arab nationalism and sought to remove the autonomy of Southerners, Southern communities had the will and ability to resist, resulting in ethnic warfare.

Sudan is not the only case in which the configuration of CCPs appears to have shaped their effects, as CCPs promoting communal inequalities in education, the administration, the legislature, and the military also contributed to ethnic warfare in Myanmar, Sri Lanka, Syria, and Lebanon (Callahan, 2003; De Silva, 1986; Fildis, 2011; Khoury, 1987; Lange et al., 2021; Selth, 1986; Smith, 1991). What appears key in these cases is that discriminatory CCPs promoted grievances that reinforced one another and communalized politics, and the combination of both contributed to conflict.

## Conclusion

In this article, we explore whether colonialism affected postcolonial patterns of ethnic conflict. For this, we engage with works comparing British and French colonialism and test claims that CCPs had long-term effects on ethnic conflict. In contrast to most past statistical analyses on the subject, we measure CCPs instead of using the identity of the colonizer as a proxy for CCPs, thereby testing claims about CCPs more directly. And whereas a few statistical analyses measure one CCP and test its relationship with ethnic conflict, we include four indicators of different CCPs, which allows us to explore the possibility that CCPs affect ethnic conflict through multiple and competing mechanisms. To analyze the different mechanisms, we create four ideal types that highlight different ways through which CCPs shape intercommunal relations, and we analyze one CCP for each type.

For insight into the effects of CCPs on ethnic conflict, we explore the odds of ethnic civil war onset and find that a highly discriminatory CCP (communal legislative representation) is associated with very high odds of ethnic civil war onset. Alternatively, we find that an empowering CCP (indirect rule) is associated with particularly low odds of ethnic civil war onset and that an accommodating CCP (vernacular education) and a differentiating CCP (communal census categories) are not related to the odds of ethnic civil war onset. We also provide evidence that the relationship between the discriminatory CCP and ethnic civil war onset changes over time, being particularly strong at and shortly after independence but weakening gradually thereafter. For the other CCPs, however, timing appears to have little impact on their effects on ethnic civil war onset. Together, the findings strongly support our hypotheses that discriminatory CCPs have positive general effects on postcolonial ethnic conflict and that these effects are strongest at independence. Contrary to our expectations, however, we provide evidence that the effects of empowering CCPs are more suppressive than mixed.

Our analysis makes several important contributions to the literatures on colonialism and ethnic civil warfare. Most notably, past analyses offer weak and inconsistent evidence about the impact of CCPs on ethnic conflict, and we find that the contrasting effects of different types of CCP help to explain previous findings. In this way, we redirect the literature by recognizing different types of CCPs and focusing on how their effects depend on their type. Relatedly, we contribute to the literature by developing different ideal types of CCPs. Past analyses focus on particular CCPs and do not consider variation in CCPs. In fact, analyses focus on specific CCPs to such an extent that we needed to create “communalizing colonial policies” as a general term to refer to this collection of related policies. Our ideal types recognize distinct ways through which CCPs affect intercommunal relations and allow researchers to explore whether different types of CCPs have different effects on ethnic conflict.

Our analysis also contributes to the literature on the timing of ethnic civil warfare. Past works provide strong evidence that the risk of ethnic warfare is particularly great during and shortly after the independence transition. Although we do not provide evidence that the general odds of ethnic civil war onset was unusually high at independence for former British and French colonies, the relationship between ethnic civil war onset and one discriminatory CCP—communal legislative representation—was particularly punctuated at independence and weakened gradually over several decades thereafter. Notably, past works argue that the risk of ethnic civil warfare is particularly great at independence because the transition from empire to nation-state promotes conflict over the character of the nation and the control of the state. Our findings, however, suggest that the extent of this risk varies from place to place and offer evidence that discriminatory CCPs help to explain this variation.

While recognizing these contributions, we end by noting the analysis’ main limitation: the inability to offer direct insight into causation. Our analysis was guided by qualitative understandings of several cases, and past case studies support our findings by offering insight into mechanisms linking discriminatory CCPs to ethnic warfare. That being said, our insight is overwhelmingly statistical, and statistical analyses provide evidence into correlation, not causation. Even more, a number of problems can affect the accuracy of statistical analyses, especially studies like our own with a relatively small number of cases (Babones, 2014; Schrank, 2013). Our findings are therefore inconclusive, and more research is needed to explore the actual processes and mechanisms underlying the complex relationship between colonialism and postcolonial ethnic warfare. Most notably, past qualitative works suggest the need to explore the impact of different discriminatory CCPs and to consider how the configurations of CCPs shape their effects. Moreover, a detailed comparative-historical analysis comparing the processes leading to positive and negative outcomes in different cases is needed to explore how indirect rule affects ethnic warfare.

## Appendix 1

### Coding of communal legislative representation and vernacular education policies

To be coded as communal legislative representation, a colony-level legislative body needed to provide special representation to at least one indigenous community at any time during the colonial period. In plantation and settler colonies with few or no indigenous peoples, we make an exception to the requirement that communities must be indigenous and decided to code cases that gave any non-European community special representation as 1. We did not, however, find any cases of communal legislative representation in these plantation and settler colonies. We recognize three situations as communal legislative representation: Either a community had reserved legislative seats, had separate electorates, or had their own legislative body. We gathered information on the presence of these types of communal legislative representation from a variety of primary and secondary sources, although information from most African cases came from Nuscheler and Ziemer (1978).

A few cases required special consideration. Colonial Malaysia had reserved seats for Chinese Malays (in addition to Indians). Most Chinese and all Indians migrated to Malaysia during the colonial period. Yet many Chinese migrated to Malaysia prior to British rule, and we consider a population “indigenous” if it resided permanently in the territory prior to colonial onset. We therefore code Malaysia as 1.

Botswana, Sierra Leone, Zambia, Uganda, and Sudan also required special consideration. In Botswana, all chiefdoms were dominated by ethnic Tswana and sent two individuals to the legislative assembly. Yet there was no requirement that the chosen representatives be Batswana, so we code the case as 0. Similarly, the legislative council in colonial Sierra Leone reserved 12 seats for paramount chiefs serving on District Councils, and we score it as 0 because any paramount chief could be selected, meaning that there was no communal requirement. In colonial Zambia, one elected representative came from the Barotseland region, which was the core of the precolonial Lozi state. Yet these members were elected, the population of this region was diverse, and candidates did not need to be Lozi. We therefore score the case as 0. Uganda is similar to Zambia in that the British reserved seats for the Buganda and Bunyoro regions, both of which had particular ethnic communities linked to precolonial states. Different from Barotseland, however, only customary authorities from the dominant ethnic communities could hold these seats, so we score the case as 1. Finally, representatives from Northern Sudan were elected, while representatives from Southern Sudan were selected from local councils. While suggesting geographic representation, the different regions coincided with major communal divisions, and the British chose to create separate systems of representation (and administration) in recognition of these differences. We therefore code the case as 1.

Singapore is a unique case in that officials selected some representatives for the legislature based on both community and occupation. In particular, colonial officials appointed three members from the Chinese and Indian Chambers of Commerce to the legislature. While suggesting communal representation, we score the case as 0 because the primary purpose of these reserved seats was to represent the business community, and business interests were organized communally in colonial Singapore.

Finally, Morocco and Tunisia are French colonial possessions, and their legislative assemblies provided Jewish communities with special representation. Unlike other cases, this special representation was ad hoc and never formalized. Because this arrangement purposefully provided a community with special representation, however, we code the cases as 1.

To categorize cases based on their educational language policies, we use Albaugh’s (2014) index on the language of primary instruction at independence for sub-Saharan African countries (and Algeria). Although this dataset does not directly measure the number of vernaculars used in primary education, we could usually decipher the number of educational vernaculars based on the index score, with Botswana, Lesotho, Sierra Leone, and Tanzania being the exceptions. For these cases, we used Albaugh (2005) to score the variable. For the remainder of British and French colonies, we gathered evidence from a variety of primary and secondary sources to identify the languages used in primary schools run by government at or shortly before independence. For all of these cases, we found clear evidence on language education policies; there were no cases requiring special consideration.

## Appendix 2. CCP scores for British and French colonies

**Table:**

Country	Legislative Representation	Multiple Education Vernaculars	Census Categories	Extent of Indirect Rule
Algeria	0	0	3	0.00
Bangladesh	1	1	5	0.50
Benin	0	0	0	0.00
Botswana	0	0	3	0.43
Burkina Faso	0	0	0	0.00
Cambodia	0	0	1	0.00
Cameroon	0	0	2	0.00
Central African Republic	0	0	3	0.00
Chad	0	0	0	0.00
Congo, Rep.	0	0	1	0.00
Cote d'Ivoire	0	0	1	0.00
Cyprus	1	1	3	0.00
Gabon	0	0	0	0.00
Gambia, The	0	0	3	0.37
Ghana	1	0	3	0.65
Guinea	0	0	2	0.00
India	1	1	5	0.49
Israel	1	1	1	0.03
Jamaica	0	0	2	0.00
Jordan	1	0	0	0.45
Kenya	0	0	2	0.59
Laos	0	0	0	0.00
Lebanon	1	0	2	0.00
Lesotho	0	0	3	0.50
Madagascar	0	0	2	0.00
Malawi	0	1	3	0.82
Malaysia	1	1	5	0.06
Mali	0	0	2	0.00
Mauritania	0	0	0	0.00
Mauritius	0	0	3	0.00
Morocco	1	1	4	0.00
Myanmar	1	1	5	0.16
Niger	0	0	0	0.00
Nigeria	1	1	4	0.93
Pakistan	1	1	5	0.50
Senegal	0	0	3	0.00
Sierra Leone	1	1	3	0.81
Singapore	0	1	4	0.00
Sri Lanka	1	1	5	0.00
Sudan	1	0	2	0.73
Swaziland	0	0	3	0.49
Syria	1	0	2	0.00
Tanzania	0	0	4	0.75
Togo	0	0	2	0.00
Trinidad and Tobago	0	0	2	0.00
Tunisia	1	0	0	0.00
Uganda	1	1	3	0.80
Vietnam	0	0	0	0.00
Zambia	0	1	2	0.60
Zimbabwe	0	1	2	0.40

CCP: communalizing colonial policy.

## Notes

1. As many recognize, however, these institutions were commonly colonial constructs, making “customary” or “traditional” misleading terms.

2. Our dataset ends in 2010, and we therefore use population in 2010 to delimit our set.

3. Data from Lange (2009) are not available for the British Mandate Territories in the Middle East. The tribal courts in colonial Palestine and Transjordan were equivalent to customary courts elsewhere, but data on the number of cases heard in tribal courts are not available. To calculate this figure for Jordan and Israel/Palestine, we therefore take the percentage of the population subject to tribal courts and divide this figure by the total population. Data are from Watkins (2014) and Bentwich (1948).

4. Although Englebert (2000) and Wimmer (2018) make opposite claims.

5. This variable measures the likelihood that two randomly selected people from the same country are from different ethnic communities, with higher scores, therefore, suggesting greater ethnic diversity. We standardize this variable in the regression models.

6. Although some previous analyses take the natural log of this variable, we do not because logging the variable does not make the relationship between this variable and logit (Pr(Ethnic war onset = 1)) any more linear. The results are not affected by this decision.

7. All controls are set to zero or the reference category. This amounts to a hypothetical French colony with no previous war onset, absence of multiple education vernaculars, and average levels of census categories, extent of indirect rule, latitude, log proportion of mountainous terrain, log population, proportion of politically excluded population, log GDP pc, and ethnic fractionalization. The overall pattern shown in the two plots are robust across different choices of control variables and their reference values.

8. Morocco and Tunisia also had communal legislative representation, but this was only temporary and non-formal.
